# 49502 CHANGES IN DEPENDENCE, WITHDRAWAL, AND CRAVING AMONG ADULT SMOKERS WHO SWITCH TO NICOTINE SALT POD-BASED E-CIGARETTES

**DOI:** 10.1017/cts.2021.622

**Published:** 2021-03-31

**Authors:** Eleanor Leavens, Nicole Nollen, Jasjit Ahluwalia, Matthew Mayo, Myra Rice, Emma Brett, Kim Pulvers

**Affiliations:** 1 University of Kansas Medical Center; 2 Brown University; 3 California State University San Marcos; 4 University of Chicago

## Abstract

ABSTRACT IMPACT: This research suggests that African American and Latinx smokers who bear a disproportionate burden of tobacco-related harms are able to switch to e-cigarettes that present reduced harm to the user due to their similar reinforcement profile to cigarettes. OBJECTIVES/GOALS: Complete switching from combustible to electronic cigarettes (ECs) reduces harm to the user. For ECs to be a viable substitute, they need to be rewarding enough for regular use, indicated by factors such as craving and dependence (reinforcement value). Little is known about short-term changes in reinforcement value across trajectories of EC use. METHODS/STUDY POPULATION: Participants were randomized 2:1 to switch to a nicotine salt pod system EC or continue smoking (assessment-only control) in a 6-week trial. 114 African American (n=60) and Latinx (n=54) smokers were randomized to receive ECs and are included in the current investigation. At week 6, participants were classified by use trajectory: exclusive smokers (n=16), exclusive EC users (n=32), or dual users (n=66). Participants reported on their EC, cigarette, and total nicotine dependence (cigarette + EC dependence), cigarette and EC use, and nicotine craving and withdrawal at baseline and week 6. Cotinine and exhaled carbon monoxide were assessed at baseline and week 6. RESULTS/ANTICIPATED RESULTS: Participants who completely switched from smoking to ECs (exclusive EC users) and those that partially switched (dual users), maintained cotinine levels (ps>.05) and showed reductions in cigarette dependence and withdrawal (ps<.01). However, exclusive EC users showed no significant changes in total nicotine dependence from baseline to week 6 (p=.123), while dual users showed increased total nicotine dependence (p<.001). Dual users displayed similar levels of EC dependence as exclusive EC users but a lesser reduction in cigarette dependence. Exclusive EC users and dual users showed reductions in craving and withdrawal from baseline to week 6. DISCUSSION/SIGNIFICANCE OF FINDINGS: This study is among the first to prospectively examine changes in dependence, craving, and withdrawal among an understudied sample of smokers making a switch attempt. Smokers who completely switch to ECs maintain nicotine levels and dependence, suggesting that they have a similar reinforcement profile to cigarettes and facilitate switching.

